# Fault Diagnosis of Brake Train Based on Multi-Sensor Data Fusion

**DOI:** 10.3390/s21134370

**Published:** 2021-06-25

**Authors:** Yongze Jin, Guo Xie, Yankai Li, Xiaohui Zhang, Ning Han, Anqi Shangguan, Wenbin Chen

**Affiliations:** Key Laboratory of Shaanxi Province for Complex System Control and Intelligent Information Processing, Xi’an University of Technology, Xi’an 710048, China; 1190313032@stu.xaut.edu.cn (Y.J.); liyankai@xaut.edu.cn (Y.L.); xhzhang@xaut.edu.cn (X.Z.); 2200320062@stu.xaut.edu.cn (N.H.); 1200313023@stu.xaut.edu.cn (A.S.); 2190321310@stu.xaut.edu.cn (W.C.)

**Keywords:** high-speed train, information fusion, fault diagnosis, parameter identification, unscented Kalman filter (UKF), expectation maximization (EM)

## Abstract

In this paper, a fault diagnosis method is proposed based on multi-sensor fusion information for a single fault and composite fault of train braking systems. Firstly, the single mass model of the train brake is established based on operating environment. Then, the pre-allocation and linear-weighted summation criterion are proposed to fuse the monitoring data. Finally, based on the improved expectation maximization, the braking modes and braking parameters are identified, and the braking faults are diagnosed in real time. The simulation results show that the braking parameters of systems can be effectively identified, and the braking faults can be diagnosed accurately based on the identification results. Even if the monitoring data are missing or abnormal, compared with the maximum fusion, the accuracies of parameter identifications and fault diagnoses can still meet the needs of the actual systems, and the effectiveness and robustness of the method can be verified.

## 1. Introduction

With the increase of speed, the reliability and safety of the train system are put forward with higher requirements. However, influenced by the potential technique abnormalities and component failures, the train system still fails frequently [1,2,3]. While these failures may not be serious in the early stages, the performance of the system has indeed been degraded [4,5]. Therefore, the early detections and identifications of any potential anomalies and failures are essential, as they avoid dangers for high-speed train operation [6,7].

The high-speed train is composed of multiple subsystems interworking with each other. A stable and reliable braking system is indispensable, to ensure a safe and comfortable operation environment. It can slow down or stop smoothly and timely when needed. Over past few decades, a large number of monitoring, diagnosis, and prediction techniques have been applied to train systems [8]. To be more specific, the fault diagnosis methods based on feature extraction, feature selection, and feature fusion have been studied in [9,10,11,12,13], and the accuracies of fault diagnosis are improved greatly by these methods. The intermittent fault detection, isolation, and diagnosis of train multi-axis speed sensors are addressed in [14,15,16], and the composite fault diagnosis of rolling equipment such as train bearings has been proposed in [17,18,19]. These above technologies have greatly improved the level of intelligence in ensuring the safe and reliable operation of trains.

In recent years, with the development of sensors, train monitoring data have become diverse. Therefore, fully mining the value of multi-source data and realizing the fault diagnosis are of great significance based on multi-sensor information fusion [20,21]. In multi-sensor fusion, centralized and distributed fusion methods are mainly used to process the monitoring data. In centralized fusion, all measured data from multiple sensors are stacked into a sensor measurement (with higher dimensions), and the specific fusion rules are not required [22,23]. However, the application of centralized fusion is limited due to some defects of its own. Therefore, various distributed fusion methods have been proposed. In distributed fusion, the state of each sensor is estimated, and then the estimations are sent to the fusion center by certain fusion criteria. For example, based on linear minimum variance, the matrix-weighted multi-sensor optimal information fusion criterion is investigated in [24,25], and distributed fusion estimations are obtained. On this basis, the multi-sensor optimal data fusion based on adaptive fading unscented Kalman filter (UKF) and unscented transform is presented in [26]. During the train operation, the integrated navigation estimation based on the GPS/INS and tachometer is considered in [27,28,29]. According to the above literature, it is not difficult to find that, compared with using only a single source of monitoring data, the fusion estimations based on multi-source data can effectively reduce the influences of various adverse factors on the analysis results.

The train-braking process is also affected by many interrelated and coupled factors, which means train braking faults cannot be accurately described and located by the existing methods and models. Hence, in order to ensure the safety of train system, the operating efficiency must be sacrificed. Even so, in extreme weather such as rainstorms and blizzards, there are still hidden dangers to the train safety. Therefore, in order to further improve the operation safety and efficiency, aiming at various brake failures under complex conditions, a train braking model is established, and a fault diagnosis strategy based on the multi-source information fusion is proposed to detect and locate train braking faults in a timely manner. The serious failures will be prevented, and useful guidance can be provided for the component maintenances and replacements.

The paper is organized as follows: In Section 2, the train air braking system is introduced, the train braking model is established, and the specific faults to be diagnosed are expounded. In Section 3, the multi-sensor data fusion strategy, multi-model state prediction, and fault diagnosis method are presented. In Section 4, the detailed experiments are carried out, and a conclusion is drawn in Section 5.

## 2. System Description and Problem Statement

In order to diagnose the faults of the train braking system quickly and accurately, in this section, a train braking model is firstly established based on the mechanism analysis, then combined with the model parameters, the train faults are analyzed, and finally the speed sensor models of the train are built.

### 2.1. Braking Modeling: Air Braking and Adhesion Braking

The actual operation of the train will be affected by the external environments. Regarding the train with complex conditions or sudden emergencies, the air emergency braking will be the last barrier to the safety of the train. The on-line detections and diagnoses of faults in the braking are helpful to prevent more serious faults and provide useful guidance information for the maintenances and replacements of components. 

**Figure 1:**
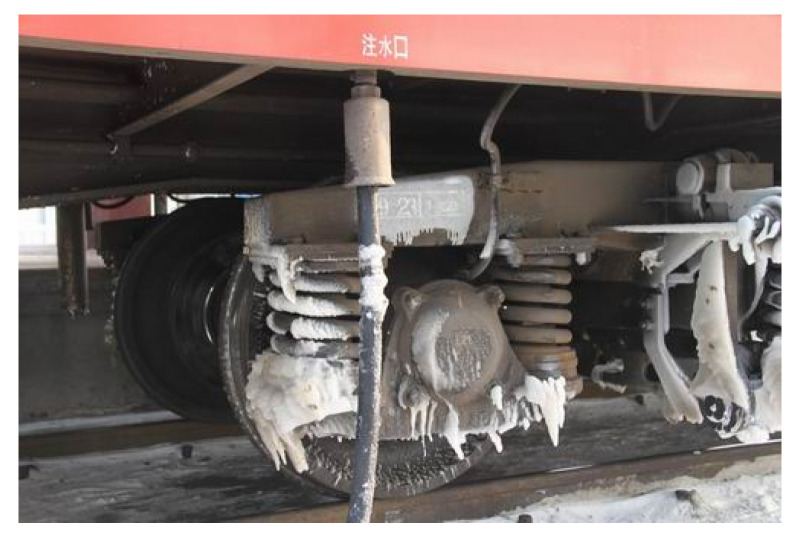
Low wheel-rail adhesion state of the train.

Considering the different characteristics of the train running on dry rail and wet rail, the influences of the wheel-rail adhesion coefficient and adhesion braking force on the train braking performance are analyzed, and the single mass point model of the train braking is established under different operation environments [30]:

(1) $$\{\begin{array}{c} v_{t+1}=\{\begin{array}{c} v_{t}-3.6T\frac{F(\mu )+R_{F}(v,C)}{M_{A}}+w_{t},F(\mu )<B\\ v_{t}-3.6T\frac{B+R_{F}(v,C)}{M_{A}}+w_{t},F(\mu )\geq B \end{array}\\ y_{t}=v_{t}+e_{t} \end{array}$$

where v is the train braking speed, C is the train current position, T and $M_{A}$ are the train sampling time and occupancy weight, F(μ) is the adhesion braking force, B is the air braking force, $R_{F}(v,C)$ is the operation resistance, $w_{t}$ is the random disturbance of speed caused by the external factors, and $e_{t}$ is the measurement error. F(μ) is jointly affected by the wheel-rail adhesion coefficient μ and the weight of the train $M_{A}$, that is:

(2) $$F(\mu )=\mu M_{A}g$$

where g is the gravitational acceleration coefficient $9.8{m/s}^{2}$. The wheel-rail adhesion coefficient is influenced by the train operation states and external environments, and its value will decrease with the increase of speed and will change with the different rail adhesion conditions. For example, as shown in Figure 1, when the train moves from dry rail to wet rail, the adhesion coefficient will decrease significantly. This will lead to the train adhesion failure, and the train system could easily lock and skid. The air braking force applied to the wheel will be invalid. As a result of this fault, the air brake of the train will shortly fail.

**Figure 2:**
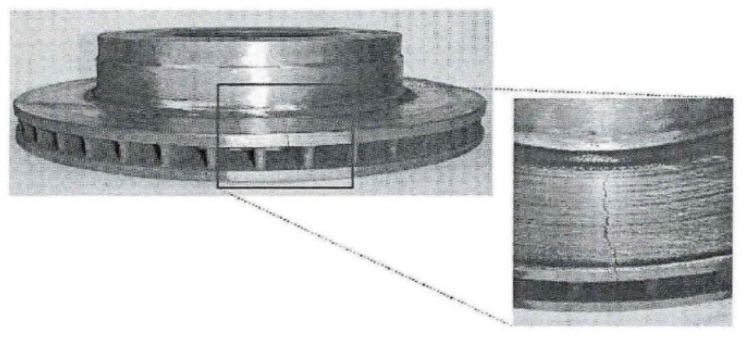
Severe wear and cracks of brake disc after long-term use.

The air braking force B is affected by several braking parameters, namely:

(3) $$B=\frac{\pi }{4}\frac{r}{R_{c}}d^{2}\times Pre\times \eta \times \gamma_{B}\times \mu_{A}\times N\times 10^{-6}$$

where d is the diameter of brake cylinder, r is the friction radius of the brake disc, $R_{c}$ is the diameter of the wheel, and N is the total number of brake pads. These parameters can be directly measured by conventional methods and are basically unchanged during the train operation, so they are treated as constants. Pre is the air pressure of the brake cylinder. During the actual emergency braking, all air pressures will be released. η is the transmission efficiency of the foundation brake device. It reflects the friction between the piston and the cylinder wall, the reverse force of the brake cylinder to release the spring, and the mechanical friction of the transmission mechanism. $\gamma_{B}$ is the braking ratio. It represents the ideal expansion multiple of the brake cylinder piston rod when the force is transferred to the brake disc. $\mu_{A}$ is the friction coefficient of the brake disc. It changes with the train running state and the brake disc material. As shown in Figure 2, when the train brake disc faults occur, such as degradations or even friction failures, it will lead to the performance decline of the train brake. Even if the brake cylinder applied the maximum air pressures, the train still cannot stop in line with the expected braking distance. Therefore, the friction coefficient has a decisive influence on the braking performance of the train, but its characteristic hidden variables mean it is difficult to directly measure with conventional methods. If the train braking failures are generated by degradations of the brake discs and the adhesion brake failures, the diagnoses of the train braking failures will become difficult.

$R_{F}(v,C)$ is the sum of the basic resistance $R_{1}(v)$ and additional resistance $R_{2}(C)$:

(4) $$R_{F}(v,C)=R_{1}(v)+R_{2}(C)$$

where $R_{1}(v)$ is positively correlated with the train running speed v:

(5) $$R_{1}(v)=M_{A}\times (c_{0}+c_{1}v+c_{2}v^{2})\times g\times 10^{-3}$$

where $c_{0}$ is the rolling resistance coefficient, $c_{1}$ is another mechanical resistance coefficient, which is directly proportional to the train speed v, and $c_{2}$ is the air resistance coefficient, which is directly proportional to the $v^{2}$. These values are usually assigned by accumulated experience.

$R_{2}(C)$ is the combination of additional resistances for ramps, curves, and tunnels:

(6) $$R_{2}(C)=R_{r}(C)+R_{c}(C)+R_{t}(C)$$

$R_{r}(C)$ is the additional resistance of the ramp at C:

(7) $$R_{r}(C)=M_{A}g\sin(\arcsin(\frac{h_{e}}{l}))$$

where $h_{e}$ and l represent the height and length of ramps, respectively.

$R_{c}(C)$ represents the additional resistance of the curve at C:

(8) $$R_{c}(C)=\{\begin{array}{cc} \frac{0.6M_{A}g}{R}, & L_{0}<L_{c}\\ \frac{0.0105M_{A}g\alpha_{L_{c}}}{L_{0}}, & L_{0}\geq L_{c} \end{array}$$

where R represents the rail radius of the curve, $L_{0}$ and $L_{c}$ are the length of the train and the curve, respectively, and $\alpha_{L_{c}}$ is the deflection angle.

$R_{t}(C)$ represents the additional tunnel resistance at C:

(9) $$R_{t}(C)=0.00013M_{A}gL$$

where L represents the length of the tunnel.

### 2.2. Speed Sensors Modeling

The train speed measurement system is composed of a variety of sensors, including an inertial navigation device (INS), a tachometer, Doppler radar, GPS, etc. Taking the CTCS3-300T train operation control system as an example, the current multi-sensor system architecture is a combination of two tachometers and two Doppler radars.

**Figure 3:**
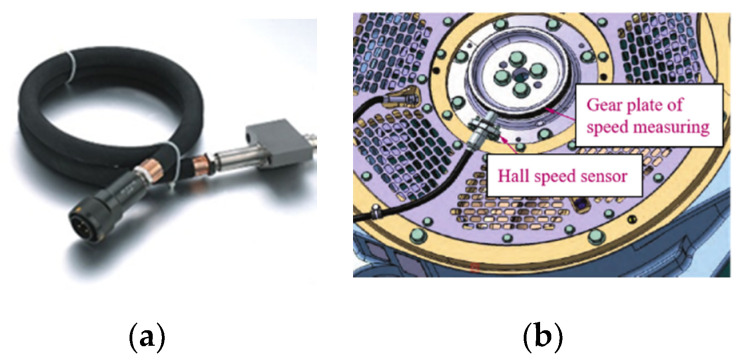
Wheel Hall speed sensor and installation example: (**a**) wheel Hall speed sensor; (**b**) installation example.

The tachometer adopts Hall’s principle. The wheel Hall speed sensor and its installation example are shown in Figure 3. When the gear disc rotates at each pitch, a counting pulse will be generated. The gear disc contains $M_{tacho}$ total of pitch, and the wheel diameter is $R_{c}$. If a total of $p_{tacho}$ pulses are received within the time interval $T_{tacho}$, the speed measurement can be expressed as follows:

(10) $$v_{tacho}=\frac{\pi R_{c}p_{tacho}}{M_{tacho}T_{tacho}}$$

It should be noted that the tachometer will be disturbed by the adhesion state of the rail surface, and the measurement accuracy will be greatly interfered when the train idling or skidding.

**Figure 4:**
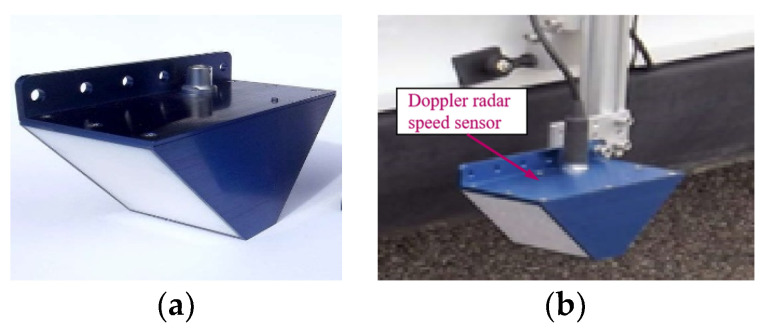
Doppler radar speed sensor and installation example: (**a**) doppler radar speed sensor; (**b**) installation example.

The Doppler effect is employed in the Doppler radar speed sensor. The Doppler radar speed sensor and its installation example are shown in Figure 4. The relative speed of train and rail is calculated by the frequency difference between the transmitted wave and ground reflection wave. The number of pulses per kilometer of the radar is recorded as $N_{dopp}$. If $p_{dopp}$ pulses are received in time interval $T_{dopp}$, the measured speed can be expressed as follows:

(11) $$v_{dopp}=\frac{1000p_{dopp}}{N_{dopp}T_{dopp}}$$

One characteristic of Doppler radar is that it is not affected by idling wheel skid and wheel diameter change. However, it requires higher installation accuracy and is more susceptible to interference than the tachometer in general.

Combined with the train braking model shown in Equation (1), the state space model of wheel speed Hall sensor is:

(12) $$\{\begin{array}{c} v_{t+1}=\{\begin{array}{c} v_{t}-3.6T\frac{F(\mu )+R_{F}(v,C)}{M_{A}}+w_{t},F(\mu )<B\\ v_{t}-3.6T\frac{B+R_{F}(v,C)}{M_{A}}+w_{t},F(\mu )\geq B \end{array}\\ p_{tacho_{t}}=\frac{M_{tacho}T_{tacho}}{\pi R_{c}}v_{t}+e_{1t} \end{array}$$

Similarly, the state space mode of Doppler radar speed sensor is:

(13) $$\{\begin{array}{c} v_{t+1}=\{\begin{array}{c} v_{t}-3.6T\frac{F(\mu )+R_{F}(v,C)}{M_{A}}+w_{t},F(\mu )<B\\ v_{t}-3.6T\frac{B+R_{F}(v,C)}{M_{A}}+w_{t},F(\mu )\geq B \end{array}\\ p_{dopp_{t}}=\frac{N_{dopp}T_{dopp}}{1000}v_{t}+e_{2t} \end{array}$$

where $e_{1}$ and $e_{2}$ are the sensor measurement errors, respectively.

## 3. Fault Diagnosis Based on Multi-Sensor Data Fusion

**Figure 5:**
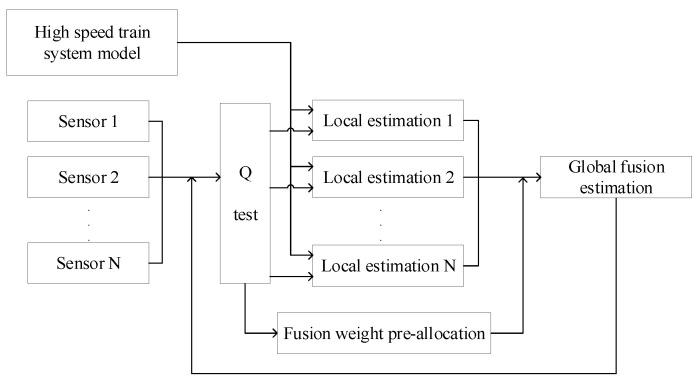
The block diagram of the proposed method.

In this section, the train braking faults are diagnosed based on the multi-sensor monitoring data. The block diagram of the proposed method is shown in Figure 5. As shown in Figure 5, the multi-sensor data are first fused by the fusion weight pre-allocation and the adaptive fading UKF. Then, the brake parameters are identified based on the improved EM (expectation maximization). Finally, the faults of train braking are diagnosed, according to the analysis of the identification results.

### 3.1. Multi-Sensor Monitoring Data Fusion

Assuming that the estimation result obtained by the *j*-th ($j=1,2,\cdots ,N_{s}$) local filter at t is ${\hat{x}}_{t}^{j}$, combined with the fusion weight pre-allocation and the linear weighted fusion criterion, the global state estimation ${\hat{x}}_{t}^{*}$ at time t of the system can be described as the following weighted sum:

(14) $${\hat{x}}_{t}^{*}=\frac{\sum_{j=1}^{N_{s}}\kappa_{t}^{j}\beta_{t}^{j}{\hat{x}}_{t}^{j}}{\sum_{j=1}^{N_{s}}\kappa_{t}^{j}\beta_{t}^{j}}$$

(15) $$\begin{array}{l} \kappa_{t}=[\kappa_{t}^{1},\kappa_{t}^{2},\cdots ,\kappa_{t}^{N_{s}}]\\ \sum_{j=1}^{N_{s}}\kappa_{t}^{j}=1,\sum_{j=1}^{N_{s}}\beta_{t}^{j}=1 \end{array}$$

where $\beta_{t}^{j}$ is the pre-allocated weight of the j-th local filter at t; ${\hat{x}}_{t}^{j}$ and $\kappa_{t}^{j}$ are the j-th local filter result and its fusion weight at t, respectively. The $\beta_{t}^{j}$, ${\hat{x}}_{t}^{j}$, and $\kappa_{t}^{j}$ will be inferred in the following sections.

### 3.2. Qualitative Analysis of Monitoring Data and Pre-Allocation of Fusion Weights

Considering the interferences of the external environments and the accuracies of the sensors it is inevitable that there will be abnormal monitoring data, missing data, and great differences in monitoring results of different types of sensors. The fusion results are affected by these abnormities seriously, thus the accuracies of diagnoses are reduced. Therefore, through in-depth mining of the information hidden under these abnormities, the faults can be diagnosed more accurately and efficiently.

In order to detect the abnormal data rapidly and accurately, the Q test is selected. Taking the speed monitoring data of sensors at t as an example, the $v_{t}^{i}$ is denoted as the speed measurement at t of the *i*-th sensor, the sequence $V_{t}$ is defined as $V_{t}=[v_{t}^{1},v_{t}^{2},\cdots ,v_{t}^{M_{s}}]$, and $M_{s}$ is the number of sensors. The monitoring data are arranged in an increasing order to get $V_{t}^{s}=[v_{t}^{1,s},v_{t}^{2,s},\cdots ,v_{t}^{M_{s},s}]$$\left(3\leq M_{s}\leq 10\right)$, then the $Q_{1}$ is formulated as follows:

(16) $$Q_{1}=\frac{\left|v_{t}^{i,s}-v_{t}^{i\pm 1,s}\right|}{v_{t}^{M_{s},s}-v_{t}^{1,s}}$$

where $v_{t}^{M_{s},s}$ and $v_{t}^{1,s}$ are the maximum and minimum of monitoring data, respectively, and $v_{t}^{i,s}$ and $v_{t}^{i\pm 1,s}$ are the measurements of the *j*-th sensor and nearest measurements, respectively. The $Q_{2}$ is determined according to the number of measurement times and the specified confidence level (e.g., 90%). If $Q_{1}>Q_{2}$, $v_{t}^{i,s}$ will be regarded as an abnormity and discarded, otherwise it should be retained. The above steps are repeated for the monitoring data until all the abnormities are eliminated and the sequence $V_{t}^{f}=[v_{t}^{1,f},v_{t}^{2,f},\cdots ,v_{t}^{N_{s},f}]\subseteq V_{t}$ is obtained.

Although there is no abnormity in $V_{t}^{f}$, the dispersion of measurements by sensors is different, which means that the external interferences are different. Therefore, in order to improve the accuracies of fusion results, a sensor real-time fusion weight pre-allocation based on distance criterion is proposed as follows:

(17) $$\beta_{t}^{i}=1-\frac{\left|v_{t}^{i,f}-{\bar{V}}_{t}^{f}\right|}{\sum_{j=1}^{N_{s}}\left|v_{t}^{j,f}-{\bar{V}}_{t}^{f}\right|}$$

(18) $${\bar{V}}_{t}^{f}=\frac{1}{N_{s}}\sum_{k=1}^{N_{s}}v_{t}^{k,f}$$

where $\beta_{t}^{i}$ is the pre-allocated fusion weight of the i-th sensor at t, and ${\bar{V}}_{t}^{f}$ is the mean measurement of sensors.

It can be inferred from Equations (17) and (18) that the number of sensors selected at each moment and the pre-allocated fusion weights are constantly changing. Compared with the existing fusion strategies with fixed weights, according to the adaptive weights determinations, the implicit value of data can be fully mined, the adverse influences on fusion filtering results can be eliminated, which contains modeling errors, measurement errors, sensor accuracies, and other adverse factors, and the real attributes of the objects to be tested can be restored as accurately as possible.

### 3.3. Multi-Sensor Data Fusion Based on Adaptive Fading UKF

The filter method and fusion strategy are the focus in this section. The fusion approach is composed of a two-layer fusion structure. In the bottom layer, the adaptive fading UKF based on Mahalanobis distance is used as the local filter [26], and the robustness of local state estimation interfered by the modeling errors is improved. At the top layer, the global state estimation based on the linear minimum variance [25] is deduced.

#### 3.3.1. Local State Estimation

Considering the Markov characteristics of Equations (12) and (13), the local filter estimator of the j-th model can be expressed as:

(19) $$\{\begin{array}{c} x_{t+1}=f(x_{t})+w_{t}\\ z_{t}=h(x_{t})+e_{t} \end{array}$$

where $x_{t}$ is the system state vector, f(⋅) is the nonlinear state function, $w_{t}$ is the state noise, which is usually regarded as the zero-mean Gaussian white noise of variance Q≥0, $z_{t}$ is the measurement of the j-th local filter, h(⋅) is the nonlinear measurement function of the j-th local filter, and $e_{t}$ is the measurement noise of the j-th local filter, which is usually assumed to be the zero-mean Gaussian white noise of variance R≥0.

#### 3.3.2. Adaptive Fading UKF Based on Mahalanobis Distance

The Mahalanobis distance of the innovation vector, which is based on hypothesis testing, is commonly adopted to identify system modeling errors for Gaussian systems. In this section, the adaptive fading UKF based on Maharanobis distance is selected as the local state estimator. By assessing the Maharanobis distance square of the innovation vector, corresponding measures are taken to improve the adaptability and robustness of UKF for multi-sensor nonlinear stochastic systems modeling. The estimation ${\hat{x}}_{t}^{j}$ of j-th sensor at t is described as follows:

**Step 1.** The initial mean square error matrix and the prior mean value of the state vector are defined as:

(20) $${\hat{x}}_{0}=E[x_{0}]$$

(21) $$P_{0}=E[(x_{0}-{\hat{x}}_{0}){\left(x_{0}-{\hat{x}}_{0}\right)}^{T}]$$

**Step 2.** Sigma sampling points of untraced transform are calculated based on sampling strategy:

(22) $$\{\begin{array}{cc} \xi_{0,t-1}={\hat{x}}_{t-1} & i=0\\ \xi_{i,t-1}={\hat{x}}_{t-1}+{\left(\sqrt{(n+\rho )P_{t-1}}\right)}_{i} & i=1,2,\cdots ,n\\ \xi_{i,t-1}={\hat{x}}_{t-1}-{\left(\sqrt{(n+\rho )P_{t-1}}\right)}_{i} & i=n+1,n+2,\cdots ,2n \end{array}$$

where n is the dimension of the system state vector, ρ is used to adjust the distance between the sampling points and the original sample points, P is the covariance matrix of state variables, and ${\left(\sqrt{(n+\rho )P_{t-1}}\right)}_{i}$ represents the i-th principal diagonal element of the square-root matrix. The weight coefficients $\omega_{i}^{1}$ and $\omega_{i}^{2}$ of the first-order and second-order statistical characteristics of the sampling points can be expressed by Equation (23):

(23) $$\omega_{i}^{1}=\omega_{i}^{2}=\{\begin{array}{cc} \frac{\rho }{n+\rho } & i=0\\ \frac{1}{2(n+\rho )} & i\neq 0 \end{array}$$

**Step 3.** The one-step prediction matrix and covariance matrix based on the Sigma sampling points are formulated as:

(24) $$\xi_{i,t|t-1}=f(\xi_{i,t-1})(i=0,1,\cdots ,2n)$$

(25) $$\{\begin{array}{l} {\hat{x}}_{t|t-1}=\sum_{i=0}^{2n}\omega_{i}^{1}\xi_{i,t|t-1}+w_{t}\\ P_{t|t-1}=\sum_{i=0}^{2n}\omega_{i}^{2}\left[\xi_{i,t|t-1}-{\hat{x}}_{t|t-1}\right]{\left[\xi_{i,t|t-1}-{\hat{x}}_{t|t-1}\right]}^{T}+Q_{t} \end{array}$$

**Step 4.** UT transformation is carried out again for the state prediction in Equation (24), and a new Sigma points set is shown in Equation (26):

(26) $$\{\begin{array}{cc} \xi_{0,t|t-1}^{'}={\hat{x}}_{t|t-1} & i=0\\ \xi_{i,t|t-1}^{'}={\hat{x}}_{t|t-1}+{\left(\sqrt{(n+\rho )P_{t|t-1}}\right)}_{i} & i=1,2,\cdots ,n\\ \xi_{i,t|t-1}^{'}={\hat{x}}_{t|t-1}-{\left(\sqrt{(n+\rho )P_{t|t-1}}\right)}_{i} & i=n+1,n+2,\cdots ,2n \end{array}$$

**Step 5.** The points set is substituted into the measurement equation, and the mean of observations is formulated as the weighted sum of the prediction.

(27) $$z_{i,t|t-1}=h(\xi_{i,t|t-1}^{’})$$

(28) $$\{\begin{array}{l} {\hat{z}}_{t|t-1}=\sum_{i=0}^{2n}\omega_{i}^{1}z_{i,t|t-1}+e_{t}=\sum_{i=0}^{2n}\omega_{i}^{1}h(\xi_{i,t|t-1}^{’})+e_{t}\\ P_{{\hat{z}}_{t|t-1}}=\sum_{i=0}^{2n}\omega_{i}^{2}[h(\xi_{i,t|t-1}^{’})-{\hat{z}}_{t|t-1}]{\left[h(\xi_{i,t|t-1}^{’})-{\hat{z}}_{t|t-1}\right]}^{T}+R_{t} \end{array}$$

Next, the state estimation covariance matrix is updated as:

(29) $$P_{{\hat{x}}_{t},{\hat{z}}_{t}}=\sum_{i=0}^{2n}\omega_{i}^{2}\left[\xi_{i,t|t-1}-{\hat{x}}_{t|t-1}\right]{\left[z_{i,t|t-1}-{\hat{z}}_{t|t-1}\right]}^{T}$$

**Step 6.** The Kalman gain is calculated, and the state and covariance are updated as follows:

(30) $$K_{t}=P_{{\hat{x}}_{t},{\hat{z}}_{t}}P_{{\hat{z}}_{t|t-1}}^{-1}$$

(31) $${\hat{x}}_{t}={\hat{x}}_{t|t-1}+K_{t}(z_{t}-{\hat{z}}_{t|t-1})$$

(32) $$P_{t}=P_{t|t-1}-K_{t}P_{{\hat{z}}_{t}}K_{t}^{T}$$

The above Steps 1–6 are the state estimation processes of classical UKF. The influences of modeling errors, measurement errors, sensor accuracies, and other adverse factors on the filtering results are not taken into account. Therefore, in this paper, the time-varying adaptive fading factor is introduced into the predicted state covariance matrix, and the influence is suppressed, which is generated by prior knowledges on the current state estimation results.

The innovation vector of the *j*-th local filter is defined as:

(33) $${\tilde{z}}_{t}=z_{t}-{\hat{z}}_{t|t-1}$$

For the nonlinear Gaussian system given in Equation (19), ${\tilde{z}}_{t}$ shall obey the zero-mean Gaussian distribution with the variance as follows:

(34) $$P_{{\hat{z}}_{t|t-1}}=\sum_{i=0}^{2n}\omega_{i}^{2}[h(\xi_{i,t|t-1}^{'})-{\hat{z}}_{t|t-1}]{\left[h(\xi_{i,t|t-1}^{’})-{\hat{z}}_{t|t-1}\right]}^{T}+R_{t}$$

The square of the Maobanobis distance of the innovation vector should obey the $\chi^{2}$ distribution of m degrees of freedom, namely:

(35) $$M_{t}^{2}={\tilde{z}}_{t}^{T}{\left(P_{{\hat{z}}_{t|t-1}}\right)}^{-1}{\tilde{z}}_{t}\sim \chi_{m}^{2}$$

where m is the measurement dimension of the j-th local filter.

In accordance with the hypothesis testing theory, for a given significance level α, the probability is denoted as:

(36) $$P(M_{t}^{2}\leq \chi_{m,\alpha }^{2})=1-\alpha (0<\alpha \leq 1)$$

where P(⋅) is the probability of the random event.

If Equation (36) is satisfied, it means that when the systems work under the optimal conditions, the nonlinear multi-sensor systems described in Equation (19) have no modeling errors, and the filtering results obtained through UKF can be identified as the local state estimations directly. Otherwise, it is determined that there are the modeling errors in the multi-sensor system (19). The time-varying adaptive fading factor is introduced into the predicted state covariance matrix, namely:

(37) $$P_{t|t-1}^{*}=\lambda_{t}(\sum_{i=0}^{2n}\omega_{i}^{2}\left[\xi_{i,t|t-1}-{\hat{x}}_{t|t-1}\right]{\left[\xi_{i,t|t-1}-{\hat{x}}_{t|t-1}\right]}^{T}+Q_{t})$$

Submitting the adaptive fading factor into the predicted state covariance matrix, Equation (36) can be rewritten as:

(38) $$g(\lambda_{t})={\tilde{z}}_{t}^{T}{\left(P_{{\hat{z}}_{t|t-1}}^{*}\right)}^{-1}{\tilde{z}}_{t}-\chi_{m,\alpha }^{2}=0$$

where $P_{{\hat{z}}_{t|t-1}}^{*}$ is the measurement prediction covariance matrix calculated by $P_{t|t-1}^{*}$.

It can be seen from Equation (38) that the solution of $\lambda_{t}$ is a problem of solving the nonlinear equation. Since the derivative of $g(\lambda_{t})$ with respect to $\lambda_{t}$ is hard to solve, the traditional Newton method is no longer applicable. In this paper, the chord secant method is used to determine the adaptive fading factor $\lambda_{t}$ by solving the nonlinear Equation (38) iteratively. The classical UKF is then completed by replacing the predicted state covariance matrix $P_{t|t-1}$ with the revised $P_{t|t-1}^{*}$ to update the local state estimation. Then, the estimation ${\hat{x}}_{t}^{j}$ of the *j*-th sensor at t is deduced as follows:

(39) $${\hat{x}}_{t}^{j}={\hat{x}}_{t}$$

The local state estimation ${\hat{x}}_{t}^{j}$ and the corresponding error covariance $P_{t}^{j}(j=1,2,\cdots ,N)$ are generated in parallel for each local filter by the above method, and then the global fusion state estimations are obtained by further fusion based on UT transform.

#### 3.3.3. Global State Estimation

Without considering the pre-allocated weights, the fusion result ${\hat{x}}_{t}^{\Delta }$ of the system’s global state at t can be expressed as:

(40) $${\hat{x}}_{t}^{\Delta }=\sum_{j=1}^{N_{s}}\kappa_{t}^{j}{\hat{x}}_{t}^{j}$$

Define and minimize the loss function shown in Equation (41):

(41) $$\Phi (\kappa )=\arg\underset{\kappa }{\min}\sum_{t=1}^{N}\left(||{\hat{x}}_{t}^{\Delta }-\sum_{j=1}^{N_{s}}\kappa_{t}^{j}{\hat{x}}_{t}^{j}|{|}_{2}^{2}\right),s.t.\sum_{j=1}^{N_{s}}\kappa_{t}^{j}=1$$

Let $\hat{X}=[{\hat{x}}_{1}^{\Delta },{\hat{x}}_{2}^{\Delta },\cdots ,{\hat{x}}_{N_{s}}^{\Delta }]$, and the loss function can be described as follows:

(42) $$\begin{array}{l} \Phi (\kappa )=\sum_{t=1}^{N}||{\hat{x}}_{t}^{\Delta }-\sum_{j=1}^{N_{s}}\kappa_{t}^{j}{\hat{x}}_{t}^{j}|{|}^{2}\\ =\sum_{t=1}^{N}||\sum_{j=1}^{N_{s}}\left({\hat{x}}_{t}^{\Delta }-{\hat{x}}_{t}^{j}\right)\kappa_{t}^{j}|{|}^{2}\\ =\sum_{t=1}^{N}||({\hat{X}}_{t}-N_{t})\kappa_{t}^{j}|{|}^{2},{\hat{X}}_{t}=[{\hat{x}}_{t}^{\Delta },\cdots ,{\hat{x}}_{t}^{\Delta }],N_{t}=[{\hat{x}}_{t}^{1},{\hat{x}}_{t}^{2},\cdots ,{\hat{x}}_{t}^{N_{s}}]\\ =\sum_{t=1}^{N}\kappa_{t}^{T}{\left({\hat{X}}_{t}-N_{t}\right)}^{T}({\hat{X}}_{t}-N_{t})\kappa_{t} \end{array}$$

The $S_{t}$ is defined as the local covariance matrix:

(43) $$S_{t}={\left({\hat{X}}_{t}-N_{t}\right)}^{T}({\hat{X}}_{t}-N_{t})$$

(44) $$\Phi (\kappa )=\sum_{t=1}^{N}\kappa_{t}^{T}S_{t}\kappa_{t}$$

The Lagrange multiplier is submitted into Φ(κ):

(45) $$L_{ag}(\kappa_{t})=\sum_{t=1}^{N}\kappa_{t}^{T}S_{t}\kappa_{t}+\lambda^{L}(\kappa_{t}^{T}I_{N_{s}}-1)$$

where the dimension of the unit vector $I_{N_{s}}$ is $N_{s}\times 1$.

The derivative of Equation (45) with respect to κ can be formulated as follows:

(46) $$\frac{\partial L_{ag}(\kappa_{t})}{\partial \kappa_{t}}=2S_{t}\kappa_{t}+\lambda^{L}I_{N_{s}}=0$$

(47) $$\kappa_{t}=\frac{S_{t}^{-1}I_{N_{s}}}{I_{N_{s}}^{T}S_{t}^{-1}I_{N_{s}}}$$

Thus, the parameters $\beta_{t}^{j}$, ${\hat{x}}_{t}^{j}$, and $\kappa_{t}^{j}$ in Equation (14) are solved. In the rest of the section, the train-braking parameters will be identified based on the fusion data, and the train-braking faults will be diagnosed.

#### 3.3.4. Fault Diagnosis Based on Fusion Data

In the previous section, the fusion filtering results of speed measurements are obtained. Based on the fusion results, the parameters of the model are identified, and the faults of the braking systems are diagnosed. Considering the parameters’ characteristics of time-varying hidden variables to be identified, an online improved expectation maximization (EM) [31,32,33] parameter identification method is proposed based on sliding window [34,35,36].

(1) Construction of conditional expectation

The train model (1) was re-expressed as follows:

(48) $$\{\begin{array}{c} v_{t+1}=F(v_{t},\theta )+\omega_{t}\\ y_{t}=H(v_{t})+e_{t} \end{array}$$

(49) $$\theta =\left[\begin{array}{c} \mu \\ \mu_{A} \end{array}\right]$$

where $y_{t}$ represents the sensor fusion result, that is, $y_{t}={\hat{x}}_{t}^{*}$, and θ is the braking parameter to be identified. Based on the Markov probability characteristics of the model, it can be inferred as follows:

(50) $$v_{t+1}\sim p_{\theta }(v_{t+1}|v_{t}),y_{t}\sim p_{\theta }(y_{t}|v_{t})$$

where $p_{\theta }(v_{t+1}|v_{t})$ represents the probability density of $v_{t+1}$ when the train state $v_{t}$ is given; $p_{\theta }(y_{t}|v_{t})$ is the probability density of $y_{t}$ given $v_{t}$.

The measurements at $t_{1}$ and $t_{2}$ are set as the head and end of the window, respectively, and the sliding length is L. The data between $t_{1}$ and $t_{2}$ are taken as the research objects, and the braking parameter θ is initialized as the estimated result ${\hat{\theta }}_{m-1}$ of the (*m* − 1)-th interval, that is:

(51) $$\theta_{m}={\hat{\theta }}_{m-1}$$

The current window output vector sequence and its likelihood function are defined as:

(52) $$Y_{t_{1}:t_{2}}=\{y_{t_{1}},\cdots ,y_{t_{2}}\}$$

(53) $$p_{\theta }(Y_{t_{1}:t_{2}})=p_{\theta }(y_{t_{1}},\cdots ,y_{t_{2}})=p_{\theta }(y_{t_{1}})\prod_{t=(t_{1}+1)}^{t_{2}}p_{\theta }(y_{t}|Y_{t_{1}:(t-1)})$$

where $p_{\theta }(y_{t}|Y_{t_{1}:(t-1)})$ represents the probability density of $y_{t}$ when the measured sequence $Y_{t_{1}:(t-1)}=\{y_{t_{1}},\cdots ,y_{t-1}\}$ is known.

Take the logarithm of both sides of Equation (53), the logarithmic of the output vectors sequence can be expressed as:

(54) $$L_{\theta }(Y_{t_{1}:t_{2}})=\ln p_{\theta }(Y_{t_{1}:t_{2}})=\ln p_{\theta }(y_{t_{1}})+\sum_{t=(t_{1}+1)}^{t_{2}}\ln p_{\theta }(y_{t}|Y_{t_{1}:(t-1)})$$

(55) $$p_{\theta }(y_{t}|Y_{t_{1}:(t-1)})=\int p_{\theta }(y_{t}|v_{t})p_{\theta }(v_{t}|Y_{t_{1}:(t-1)})dv_{t}$$

where $p_{\theta }(y_{t}|v_{t})$ represents the probability density of $y_{t}$ when the $v_{t}$ is given. Due to the existence of unmeasurable parameters to be identified in the model, the train state sequence $V_{t_{1}:t_{2}}=\{v_{t_{1}},\cdots ,v_{t_{2}}\}$ is regarded as an incomplete measurement. The joint probability density function of all data is derived from the $V_{t_{1}:t_{2}}$ and train output observations $Y_{t_{1}:t_{2}}$.

(56) $$\begin{array}{l} L_{\theta }(V_{t_{1}:t_{2}},Y_{t_{1}:t_{2}})=\ln p_{\theta }(V_{t_{1}:t_{2}},Y_{t_{1}:t_{2}})=\ln p_{\theta }(V_{t_{1}:t_{2}}|Y_{t_{1}:t_{2}})+\ln p_{\theta }(Y_{t_{1}:t_{2}})\\ =\ln p_{\theta }(v_{t_{1}})+\sum_{t=t_{1}}^{t_{2}-1}\ln p_{\theta }(v_{t+1}|v_{t})+\sum_{t=t_{1}}^{t_{2}}\ln p_{\theta }(y_{t}|v_{t}) \end{array}$$

It is assumed that the current estimation of train brake coefficient is $\theta_{k}$, and the expectation $E(\theta ,\theta_{k})$ of $L_{\theta }(V_{t_{1}:t_{2}},Y_{t_{1}:t_{2}})$ is described as follows:

(57) $$E(\theta ,\theta_{k})=\int \ln p_{\theta }(V_{t_{1}:t_{2}},Y_{t_{1}:t_{2}})\times p_{\theta_{k}}(V_{t_{1}:t_{2}}|Y_{t_{1}:t_{2}})dV_{t_{1}:t_{2}}=I_{1}+I_{2}+I_{3}$$

(58) $$\begin{array}{l} I_{1}=\int \ln p_{\theta }(v_{t_{1}})p_{\theta_{k}}(v_{t_{1}}|Y_{t_{1}:t_{2}})dv_{t_{1}}\\ I_{2}=\sum_{t=t_{1}}^{t_{2}-1}\iint \ln p_{\theta }(v_{t+1}|v_{t})p_{\theta_{k}}(v_{t+1},v_{t}|Y_{t_{1}:t_{2}})dv_{t}v_{t+1}\\ I_{3}=\sum_{t=t_{1}}^{t_{2}}\int \ln p_{\theta }(y_{t}|v_{t})p_{\theta_{k}}(v_{t}|Y_{t_{1}:t_{2}})dv_{t} \end{array}$$

where $I_{1}$,$I_{2}$, and $I_{3}$ are related to conditionally smooth densities $p_{\theta_{k}}(v_{t_{1}}|Y_{t_{1}:t_{2}})$, $p_{\theta_{k}}(v_{t+1},v_{t}|Y_{t_{1}:t_{2}})$, and $p_{\theta_{k}}(v_{t}|Y_{t_{1}:t_{2}})$, respectively. Considering that there are unmeasurable variables in the train state, the analytical solution by integral method is invalid, so the integration is solved by the particle filter and particle smoothing.

(2) Maximization of conditional expectation

From Equations (57) and (58), it can be seen that the relationship is nonlinear and non-convex between the estimation of conditional expectation $\hat{E}(\theta ,\theta_{k})$ and train braking parameters, and its closed solution is hard to solve. Therefore, the gradient optimization is adopted to find θ, which makes $\hat{E}(\theta ,\theta_{k})$ the maximum [37]. The partial derivative of $\hat{E}(\theta ,\theta_{k})$ with respect to θ is as follows:

(59) $$\frac{\partial }{\partial \theta }\hat{E}(\theta ,\theta_{k})=\frac{\partial {\hat{I}}_{1}}{\partial \theta }+\frac{\partial {\hat{I}}_{2}}{\partial \theta }+\frac{\partial {\hat{I}}_{3}}{\partial \theta }$$

The gradient method is adopted for parameter iterative optimization. When the estimation accuracy of braking parameter meets $||\theta_{k+1}-\theta_{k}||\leq \varepsilon$, the iteration terminates, and $\theta_{k+1}$ is regarded as the final estimation result ${\hat{\theta }}_{m}$ of the m-th window. Otherwise, the iteration continues.

(60) $${\hat{\theta }}_{m}=\theta_{k+1}$$

${\hat{\theta }}_{m}$ is regarded as the parameter identification result at the midpoint of $t_{1}$ and $t_{2}$.

(61) $${\hat{\theta }}_{(t_{1}+t_{2})/2}={\hat{\theta }}_{m}$$

## 4. Analysis of Simulation Results

In order to verify the validity of the diagnostic method presented in this paper, a CRH3 high-speed train with four motions and four tows is selected for the simulation experiment.

### 4.1. Train Parameters Descriptions

In the train braking simulation, the main parameters of the braking device are shown in Table 1.

The main parameters of the speed sensors are shown in Table 2.

The wheel-rail adhesion coefficient is selected by the actual experience, which can be divided into dry rail and wet rail adhesion coefficient by the different rail states, as follows:

Dry rail:

(62) $$\mu =0.06+\frac{46.6}{260+v}$$

Wet rail:

(63) $$\mu =0.04+\frac{13.7}{120+v}$$

As can be seen from the above equation, the adhesion coefficient of different rail states is different, and it is inversely proportional to the train speed. When the train is running on a dry rail, the system fault that results in poor braking performance can be easily diagnosed. However, when the train is in emergency braking on wet rail, the adhesion coefficient is difficult to be accurately measured, which makes it difficult to distinguish whether the braking performance degradations are caused by braking system failures, train adhesion failures, or both. Therefore, the following four typical cases of the train braking will be taken as examples for simulations.

### 4.2. Four Typical Cases

#### 4.2.1. Adhesion Normal and Braking Normal

**Figure 6:**
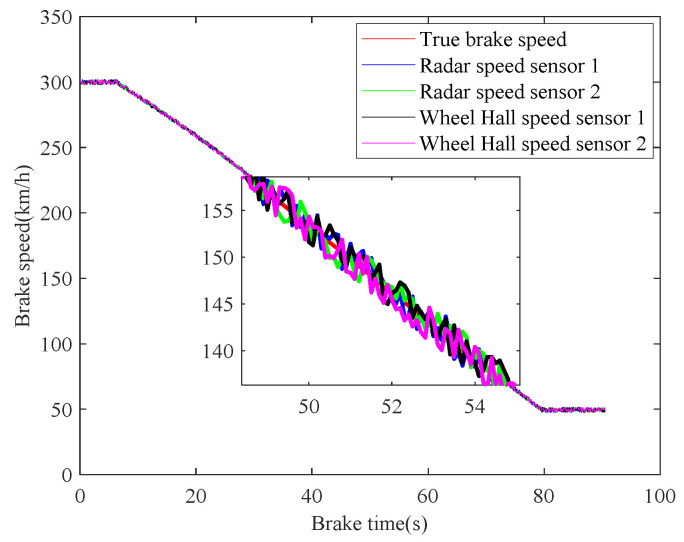
Train braking speed measurements.

**Figure 7:**
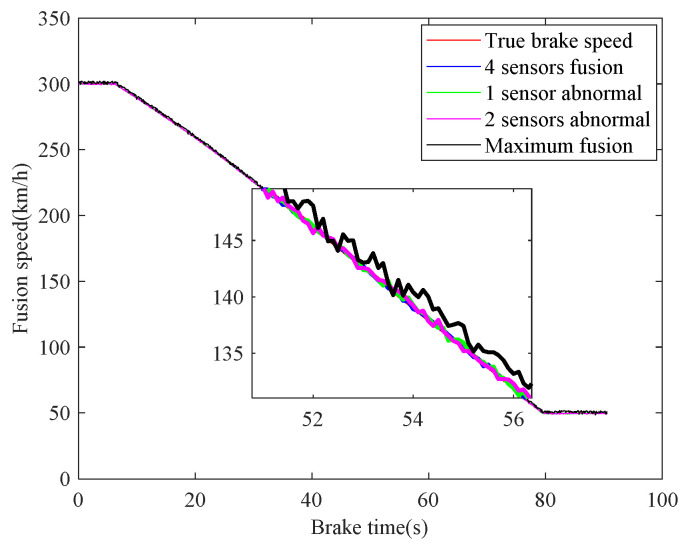
Fusion of speed sensors.

In case 1, the braking characteristics of the train are studied with normal adhesion. The braking speed measurements are shown in Figure 6. The red curve is the true braking speed, and the other four curves represent the four sensors’ speed measurements. As can be seen from the Figure 6, in the emergency braking, the train speed decreases from 300 km/h to 50 km/h, the braking time is 73.3 s, and the average deceleration speed is 0.9474 m/s^2^. The train speed fusion results are shown in Figure 7. The red curve is the real speed of the train, the blue curve is the fusion result of the four speed sensors, the green curve is the fusion result of the three speed sensors in the case of abnormal or lost data caused by sensor failures or network transmission failures, the magenta curve is similarly the fusion result of the two speed sensors, and the black curve is the maximum speed fusion result equipped in the train’s on-board ATP. It can be seen from the Figure 7 that the multiple speed measurements can be fused effectively by the method proposed in this paper. Affected by the missing or abnormal sensors measurements, in the case of meeting the actual accuracy requirements of the system, the fusion accuracy of four speed sensors is slightly higher than the other two cases. However, when comparing with the maximum fusion strategy equipped in the train’s on-board ATP, the fusion accuracy of the proposed method is obviously higher.

**Figure 8:**
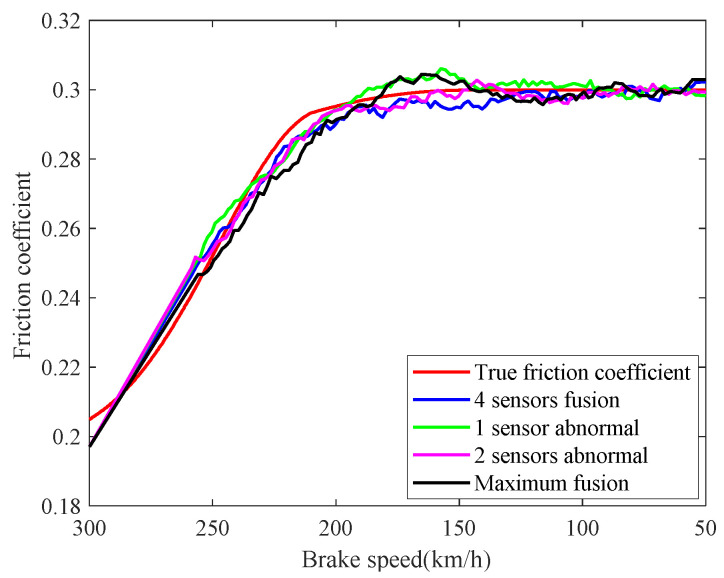
Identifications of friction coefficient.

**Figure 9:**
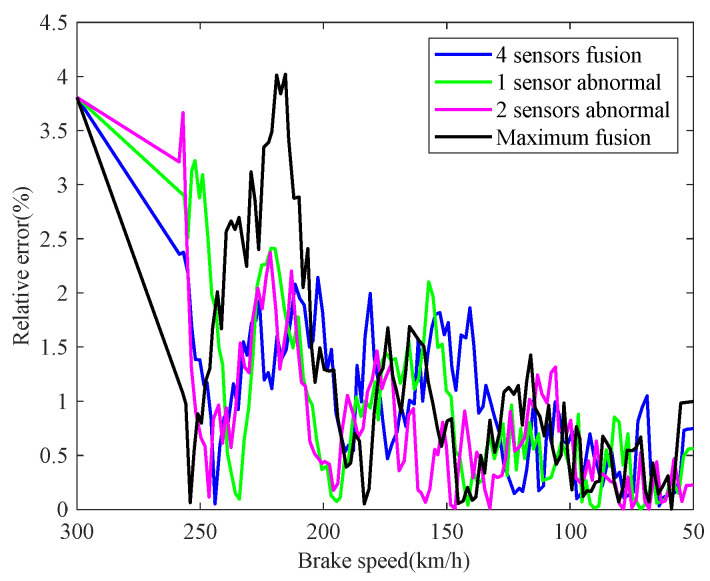
Identification relative errors of friction coefficient.

The real-time identification results of the friction coefficient of the brake disc are shown in Figure 8. The red curve is the real value of the friction coefficient, and the other curves correspond to the friction coefficient identification values based on the speed fusion results in Figure 7. It can be seen from Figure 8, based on the fusion speed, the unobservable time-varying friction coefficient can be identified accurately, then the real-time braking performance of the train brake disc can be grasped timely. The identification result of the black curve differs greatly from the real value universally, because the speed obtained based on the maximum fusion strategy is higher than the real speed. The identification relative errors of friction coefficient are shown in Figure 9.

The speed fusion errors and parameter identification errors are shown in Table 3. It can be seen from the table that the speed fusion error and friction coefficient identification error of the method proposed in this paper are ±1.7472% and ±2.4891%, respectively, and the speed fusion error and friction coefficient identification error of the maximum fusion are ±2.5627% and ±2.4963% respectively. It can be concluded, even for the maximum fusion data with large error, that the real braking performance of the brake disc can still be obtained effectively and accurately by the fusion identification framework proposed in this paper.

#### 4.2.2. Adhesion Normal but Brake Degradation

**Figure 10:**
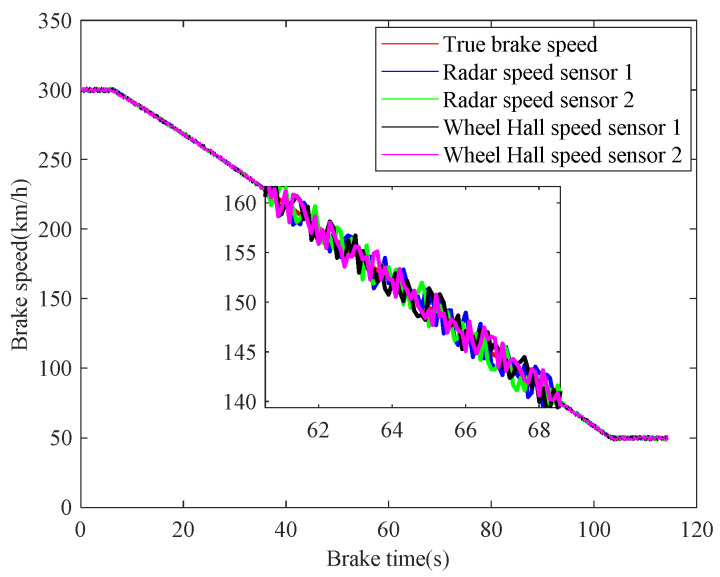
Train braking speed measurements.

**Figure 11:**
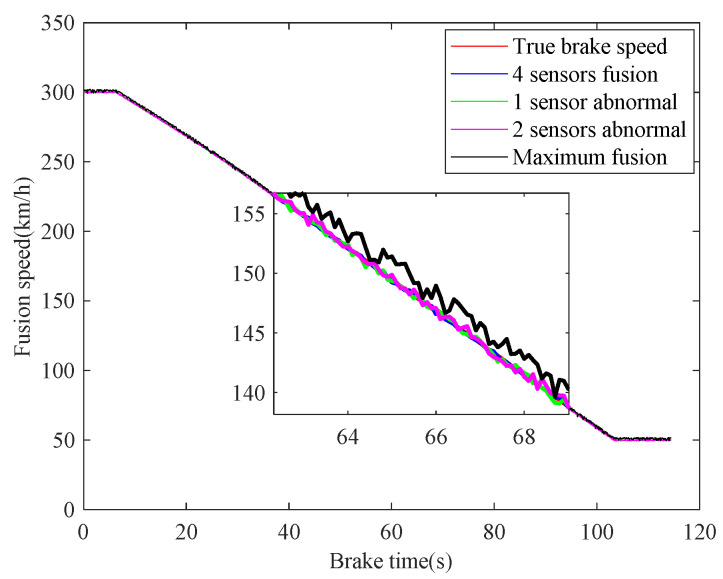
Fusion of speed sensors.

In case 2, the braking characteristics of the train under normal rail adhesion are studied. Different from case 1, the braking performance of the train declined, and the change of braking characteristics of the brake disc is paid more attention in case 2. The braking speed measurements are shown in Figure 10. The red curve is the true braking speed, and the other four curves represent the sensors speed measurements. It can be seen from the Figure 10 that the train speed is reduced from 300 km/h to 50 km/h, the braking time is 97.1 s, and the average deceleration is 0.7152 m/s^2^. Compared with the normal braking in case 1, the braking time increases by 23.8 s and 32.47%, and the average deceleration speed decreases by 0.2322 m/s^2^ and 24.51% in case 2. Therefore, it is considered that the braking performance of the train decreases significantly. Similar to Figure 7, the fusion results of train speed are shown in Figure 11, which are based on the proposed method and the maximum fusion. It can be seen from Figure 11 that the multi-sensor train speed measurements can be effectively fused by the proposed method. Under meeting the actual accuracy requirements of the system, the fusion accuracy of four sensors speed measurements is slightly higher than that of the other two cases; however, compared with the maximum fusion results, the fusion accuracy of the proposed method is higher.

**Figure 12:**
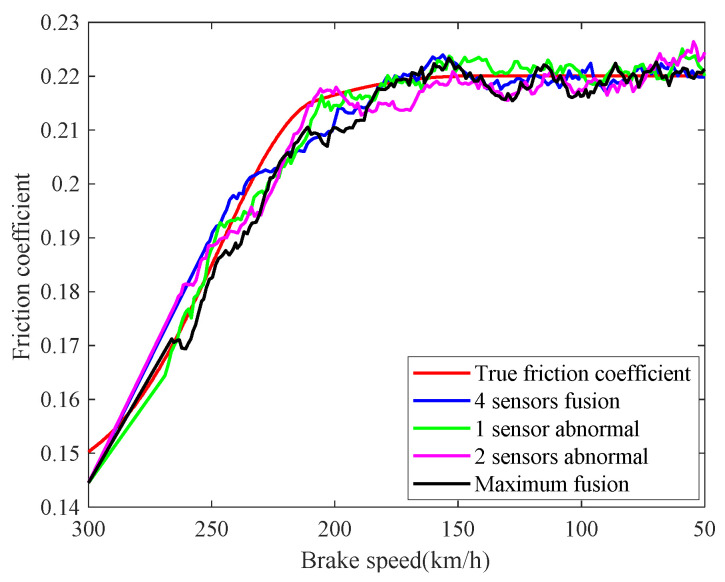
Identifications of friction coefficient.

**Figure 13:**
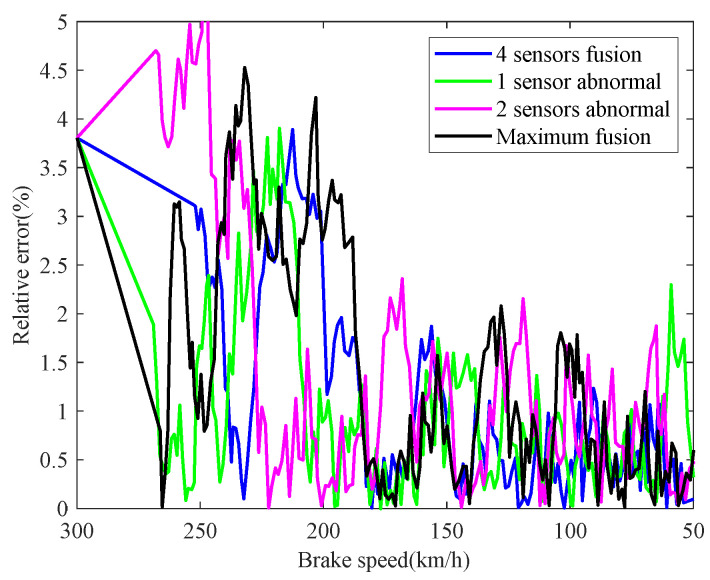
Identification relative errors of friction coefficient.

The real-time identification results of the friction coefficient of the brake disc are shown in Figure 12. The red curve is the real value of the friction coefficient, and the other curves correspond to the friction coefficient identifications based on the speed fusion results in Figure 11. Compared with the normal braking parameter identification curves in Figure 8, it can be seen that the friction coefficient of the brake disc in case 2 significantly decreased. In the train braking, the speed decreases from 300 km/h to 50 km/h, and the friction coefficient of the brake disc gradually stabilizes at 0.22, which decreases by 0.08 and 26.67%, compared with 0.3 in Figure 8. Combined with the current braking capacity of the brake disc and the train braking standard, the continued use of the brake disc will pose a threat to the train operation safety. Therefore, when the brake disc is diagnosed with a brake fault, it should be replaced in a timely manner. The identification relative errors of friction coefficient are shown in Figure 13.

The speed fusion errors and parameter identification errors are shown in Table 4. It can be seen from the table that the speed fusion error and friction coefficient identification error of the proposed method are ±1.5152% and ±2.4937%, respectively. The speed fusion error and friction coefficient identification error of maximum fusion are ±2.3187% and ±2.4987%, respectively. Even for the maximum fusion data with large errors, the real braking performance of the brake disc can be still identified effectively and accurately, and the robustness is verified of the proposed fusion identification framework.

#### 4.2.3. Adhesion Failure but Braking Normal

**Figure 14:**
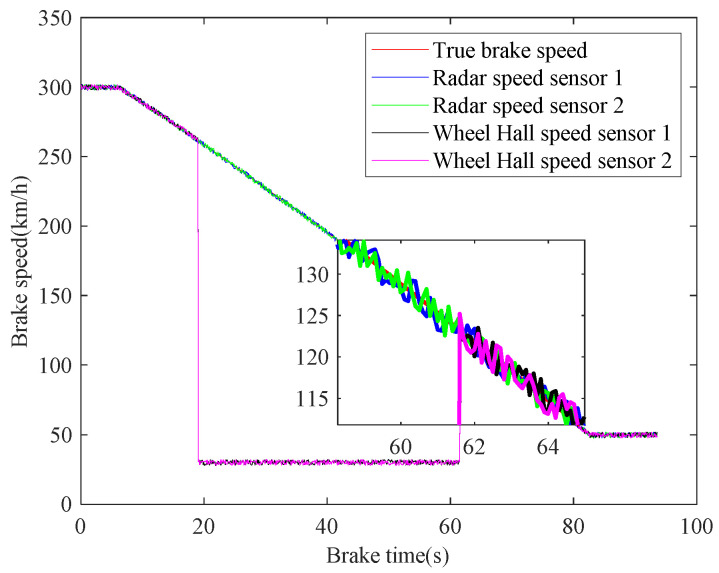
Train braking speed measurements.

**Figure 15:**
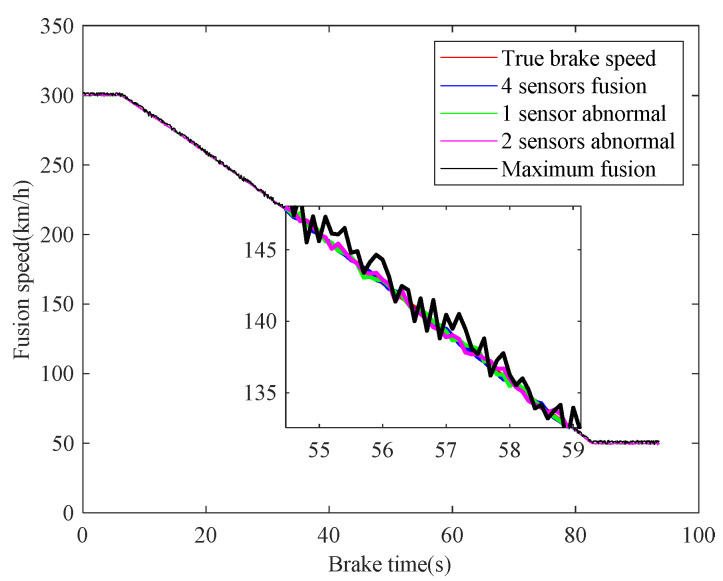
Fusion of speed sensors.

In case 3, the braking characteristics of trains with rail adhesion failure are studied. The braking speed measurements are shown in Figure 14. The red curve represents the actual braking speed, the blue and green curves represent the radar speed measurements, and the black and magenta curves represent the wheel-rail Hall sensors speed measurements. As can be seen from Figure 14, during the whole braking process, the radar speed measurement curves are close to the real value at all times, while the wheel Hall speed measurement curves plummet in 19.0–61.6 s (261.60 km/h–124.74 km/h), and the two Hall speed measurement results are similar. Combined with the Hall speed measuring principle, it can be inferred that the braking process from 300km/h to 50 km/h is a composite braking, in which 261.60 km/h–124.74 km/h is adhesive braking, and 300 km/h–261.60 km/h and 124.74 km/h–50 km/h are air emergency braking. The braking time of the whole braking process is 77.7 s, and the average deceleration is 0.8938 m/s^2^. The fusion results of speed are shown in Figure 15, which are fused based on the proposed fusion method and the maximum fusion. It can be seen from Figure 15 that the speed measurements of multiple trains can be effectively fused by the proposed paper. Compared with the maximum fusion results, the fusion accuracy of the proposed fusion method is still higher.

**Figure 16:**
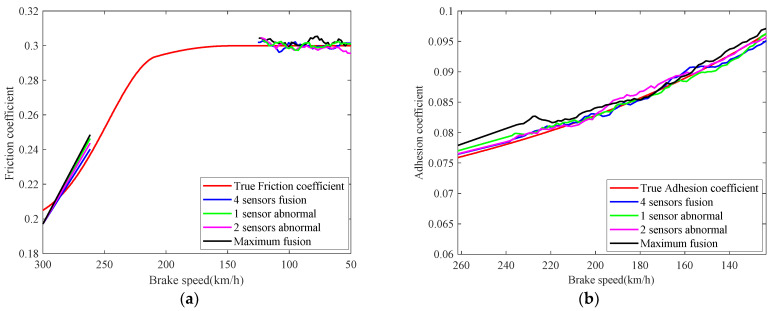
Identifications of friction coefficient and adhesion coefficient: (**a**) friction coefficient; (**b**) adhesion coefficient.

**Figure 17:**
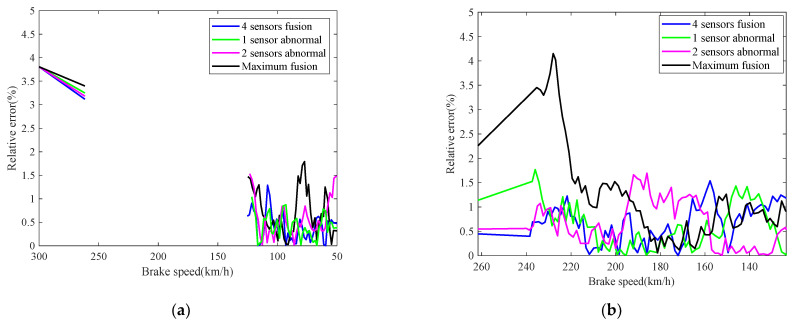
Identification relative errors of friction coefficient and adhesion coefficient: (**a**) friction coefficient; (**b**) adhesion coefficient.

Considering the model switching in the train composite braking, the friction coefficient of the brake disc is identified in the 300 km/h–261.60 km/h and 124.74 km/h–50 km/h range, and the wheel-rail adhesion coefficient is identified in the 261.60 km/h–124.74 km/h range. The real-time identification results of the friction coefficient and the adhesion coefficient are shown in Figure 16. The red lines are the real values of the friction coefficient and adhesion coefficient, respectively. The other curves correspond to the identifications of the friction coefficient and adhesion coefficient in Figure 15, respectively. As can be seen from Figure 16, the hidden parameters in the train jump model can be accurately estimated by the fusion identification framework proposed in this paper. Then, it can be concluded that the intermittent braking failure is not caused by the decrease of friction performance of brake disc fault, but the adhesion failure of adhesion coefficient between wheel and rail on the wet rail. The identification of the relative errors of the friction coefficient and the adhesion coefficient are shown in Figure 17.

The speed fusion errors and parameter identification errors are shown in Table 5. It can be seen from the table that the speed fusion error, friction coefficient, and adhesion coefficient identification error of the proposed method are ±1.6742%, ±2.2473%, and ±1.7267%, respectively, and the speed fusion error, friction coefficient, and adhesion coefficient identification error of the maximum fusion are ±2.4659%, ±2.3682%, and ±1.7431%, respectively. Even for the maximum fusion data with large errors, the braking performance of the brake disc and the real-time wheel-rail adhesion state can be more effectively and accurately obtained.

#### 4.2.4. Adhesion Failure and Brake Degradation

**Figure 18:**
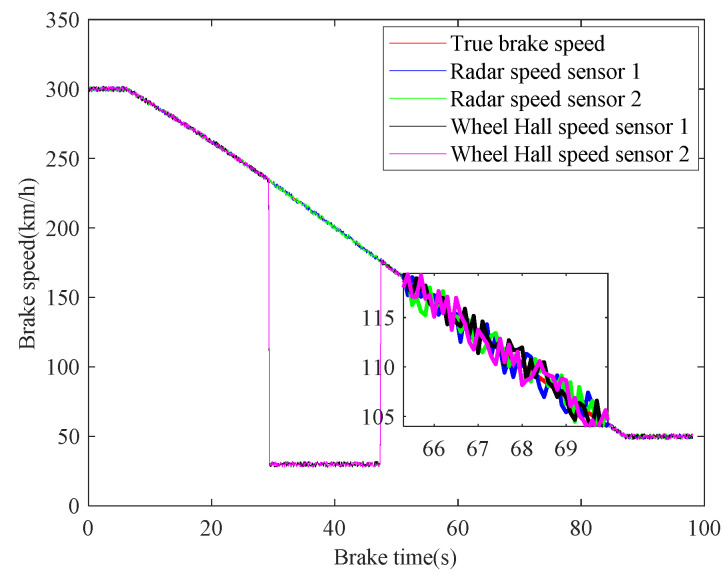
Train braking speed measurements.

**Figure 19:**
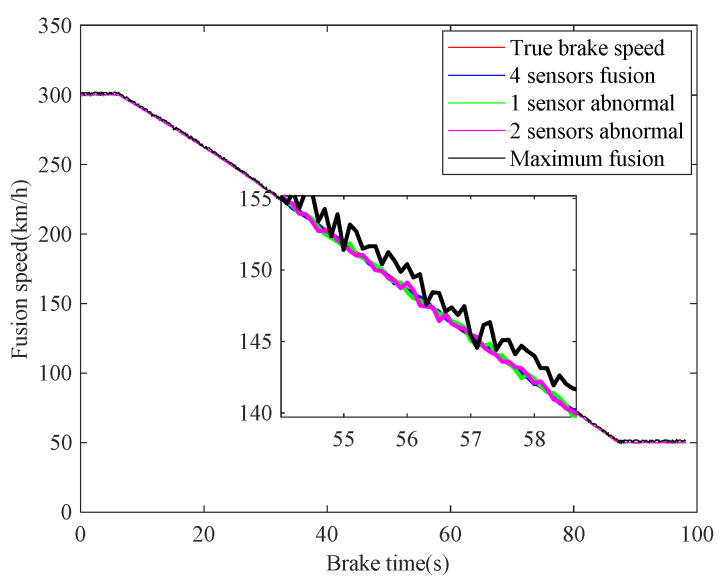
Fusion of speed sensors.

In case 4, the braking characteristics with adhesion failures are also studied. The braking speed measurements are shown in Figure 18. The red curve represents the true braking speed of the train, the blue and green curves represent the radar speed measurement curves, and the black and magenta curves represent the wheel-rail Hall speed measurement curves. Similar to Figure 14, there is also a sharp drop in the two Hall speed measurements in Figure 18, which indicates that the braking process of the train is composite braking, 234.47 km/h–185.38 km/h is adhesive braking, and 300 km/h–234.47 km/h and 185.38 km/h–50 km/h are air emergency braking. In the whole braking process, the braking duration is 80.9 s and the average deceleration is 0.8544 m/s^2^. Compared with case 3, in case 4, the braking time increases by 3.2 s and 3.96%, and the average deceleration decreases by 0.0354 s and 3.96%. Therefore, it is considered that the train braking performance similarly decreases. The fusion results of train speed are shown in Figure 19, which are fused based on the proposed fusion method and the maximum fusion. It can be seen from Figure 19 that the measurements of multiple sensors can be fused effectively. Compared with the maximum fusion, the fusion accuracy of the proposed fusion method is still higher.

**Figure 20:**
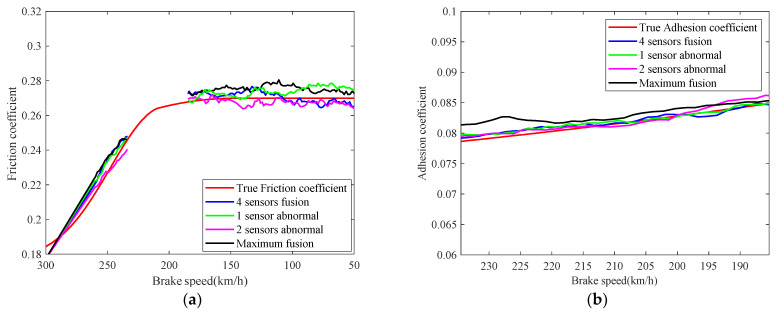
Identifications of friction coefficient and adhesion coefficient: (**a**) friction coefficient; (**b**) adhesion coefficient.

**Figure 21:**
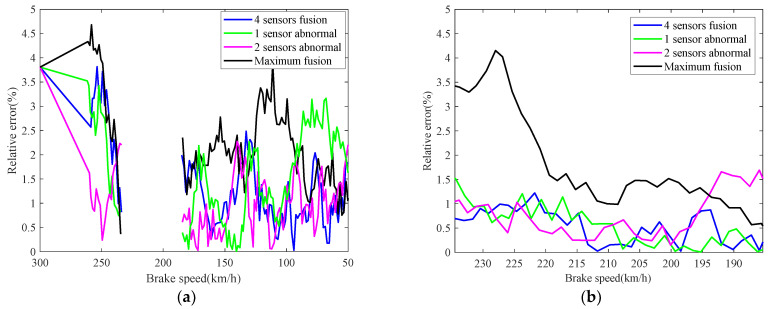
Identification relative errors of friction coefficient and adhesion coefficient: (**a**) friction coefficient; (**b**) adhesion coefficient.

The real-time identification results of the friction coefficient and the wheel-rail adhesion coefficient are shown in Figure 20. The red lines are the true values of the friction coefficient and the adhesion coefficient, respectively. The other curves correspond to the identifications of the friction coefficient and the adhesion coefficient based on the speed fusion results in Figure 19. As can be seen from Figure 20, the wheel-rail adhesion characteristics remain unchanged, but comparing with the normal friction coefficient in case 1, the friction performance of the brake discs in case 4 decreases significantly. The identification of relative errors of the friction coefficient and adhesion coefficient are shown in Figure 21.

The speed fusion errors and parameter identification errors are shown in Table 6. It can be seen from the table that the speed fusion error, identification errors of the friction coefficient, and the adhesion coefficient of the proposed method are ±1.4364%, ±2.4967%, and ±1.6733%, respectively. The speed fusion error, identification errors of the friction coefficient, and the adhesion coefficient of the maximum fusion are ±2.3751%, ±2.4989%, and ±1.7875%, respectively. Even for the maximum fusion data with large errors, the braking performance of the brake disc and the real-time wheel-rail adhesion state can be more effectively and accurately obtained.

## 5. Conclusions and Prospect

Based on the train braking mechanism and the actual operating environments, the train emergency braking model based on the operation environment is established. Aiming at the composite braking faults of the train, the fusion weights pre-allocation and linear weighted fusion criteria are constructed, and the braking modes and braking parameters are identified based on the improved online maximum expectation, and the faults of the train braking system are diagnosed. The simulation results show that the braking parameters of systems can be effectively identified, the identification relative errors of the speed, friction coefficient, and adhesion coefficient are no more than 1.8%, 2.5%, and 1.75%, respectively, which meet the actual requirements of the brake system. Furthermore, the single fault and composite fault of the train braking system can be accurately diagnosed, even if the monitoring data are disturbed or missing. The effectiveness and robustness of the proposed method are verified. It can be concluded that the application of this method will greatly improve the accuracy of train operation monitoring and fault diagnosis, reduce the daily maintenance cost, and ensure train safety.

In future research, the algorithm will be improved and optimized to adjust the size of the sliding window adaptively, and the speed and accuracy of parameter estimation will be further improved. In the future, more types of sensor data will be fused, and a faster fusion algorithm will also be developed. Different trains and types of faults will also be considered.

## Figures and Tables

**Table 1:** Braking parameters of CRH3.

Braking Parameters	Value
Total Weight of Train/(t)	536
Maximum Operating Speed/(km/h)	350
Continuous Operating Speed/(km/h)	300
Brake Cylinder Diameter/(mm)	203
Air Pressure of Brake Cylinder/(kpa)	410
Transmission Efficiency	0.85
Braking Ratio	2.55
Friction Coefficient	0.28
Brake Disc Friction Radius	297.6
Wheel Rolling Radius/(mm)	460

**Table 2:** Speed sensors parameters.

Wheel Hall Speed Sensor	Value	Doppler Radar Speed Sensor	Value
Pitch	7.85	Range Resolution/(mm/pulse)	4
Gear module	2.5	Number of Pulses/(km)	250,000
Operation Temperature/(°C)	20~80	Operation Temperature/(°C)	−20~70

**Table 3:** Speed fusion errors and parameter identification errors.

	Fusion Method	4 Sensors Fusion	3 Sensors Fusion	2 Sensors Fusion	Maximum Fusion
Relative Error					
Brake Speed Fusion		±0.5313%	±1.3412%	±1.7472%	±2.5627%
Identification of Friction Coefficient		±2.2684%	±2.4832%	±2.4891%	±2.4963%

**Table 4:** Speed fusion errors and parameter identification errors.

	Fusion Method	4 Sensors Fusion	3 Sensors Fusion	2 Sensors Fusion	Maximum Fusion
Relative Error					
Brake Speed Fusion		±0.6049%	±1.2527%	±1.5152%	±2.3187%
Identification of Friction Coefficient		±2.2258%	±2.4863%	±2.4937%	±2.4987%

**Table 5:** Speed fusion errors and parameter identification errors.

	Fusion Method	4 Sensors Fusion	3 Sensors Fusion	2 Sensors Fusion	Maximum Fusion
Relative Error					
Brake Speed Fusion		±0.5657%	±1.5629%	±1.6742%	±2.4659%
Identification of Friction Coefficient		±1.8477%	±1.9337%	±2.2473%	±2.3682%
Identification of Adhesion Coefficient		±1.5391%	±1.6538%	±1.7267%	±1.7431%

**Table 6:** Speed fusion errors and parameter identification errors.

	Fusion Method	4 Sensors Fusion	3 Sensors Fusion	2 Sensors Fusion	Maximum Fusion
Relative Error					
Brake Speed Fusion		±0.6116%	±1.2220%	±1.4364%	±2.3751%
Identification of Friction Coefficient		±2.1127%	±2.3431%	±2.4967%	±2.4989%
Identification of Adhesion Coefficient		±1.5087%	±1.6325%	±1.6733%	±1.7875%

## Data Availability

The data presented in this study are available on request from the corresponding author. The data are not publicly available due to the confidentiality.
